# Estimating cumulative point prevalence of rare diseases: analysis of the Orphanet database

**DOI:** 10.1038/s41431-019-0508-0

**Published:** 2019-09-16

**Authors:** Stéphanie Nguengang Wakap, Deborah M. Lambert, Annie Olry, Charlotte Rodwell, Charlotte Gueydan, Valérie Lanneau, Daniel Murphy, Yann Le Cam, Ana Rath

**Affiliations:** 1 Inserm, US14-Orphanet, Paris, France; 2 Orphanet Ireland, National Rare Diseases Office, Mater Misericordiae University Hospital, Dublin, Ireland; 3 Eurordis - Rare Diseases Europe, Plateforme Maladies Rares, Paris, France

**Keywords:** Health policy, Economics

## Abstract

Rare diseases, an emerging global public health priority, require an evidence-based estimate of the global point prevalence to inform public policy. We used the publicly available epidemiological data in the Orphanet database to calculate such a prevalence estimate. Overall, Orphanet contains information on 6172 unique rare diseases; 71.9% of which are genetic and 69.9% which are exclusively pediatric onset. Global point prevalence was calculated using rare disease prevalence data for predefined geographic regions from the ‘Orphanet Epidemiological file’ (http://www.orphadata.org/cgi-bin/epidemio.html). Of the 5304 diseases defined by point prevalence, 84.5% of those analysed have a point prevalence of <1/1 000 000. However 77.3–80.7% of the population burden of rare diseases is attributable to the 4.2% (*n* = 149) diseases in the most common prevalence range (1–5 per 10 000). Consequently national definitions of ‘Rare Diseases’ (ranging from prevalence of 5 to 80 per 100 000) represent a variable number of rare disease patients despite sharing the majority of rare disease in their scope. Our analysis yields a conservative, evidence-based estimate for the population prevalence of rare diseases of 3.5–5.9%, which equates to 263–446 million persons affected globally at any point in time. This figure is derived from data from 67.6% of the prevalent rare diseases; using the European definition of 5 per 10 000; and excluding rare cancers, infectious diseases, and poisonings. Future registry research and the implementation of rare disease codification in healthcare systems will further refine the estimates.

## Introduction

Rare diseases (RDs) are numerous, heterogeneous in nature, and geographically disparate. Few are preventable or curable, most are chronic and many result in early death. Despite their heterogeneity, RDs share commonalities linked to their rarity that necessitates a comprehensive public health approach [1, 2]. The challenges arising from their low prevalence: a lack of knowledge and scarcity of expertise as well as their chronic, degenerative, and life-threatening nature, have led to RDs emerging as a public health priority in Europe [3–6].

While there is no universal definition of RDs [7], the concept of RDs in the current political and legislative framework is closely linked to a definition according to point prevalence, and existing definitions are explicitly or implicitly based on a prevalence threshold. The Council of Ministers of the European Union (EU), has suggested that between 6 and 8% of the European population could be affected by a RD in the course of their lives [8]. In the EU, the definition of RDs was established in EU Regulation on orphan medicinal products (1999) as conditions whose prevalence is not more than 50 per 100 000 [4]. The American Orphan Drug Act (1983) defined RDs as disorders affecting <200 000 persons in the country, translating to a prevalence of 86 per 100 000 at that time [9]. Other national definitions translate to prevalence ranging from 5 to 76 per 100 000 [10–14]. Point prevalence is the most appropriate indicator for RDs as it provides a measurement of the population burden of disease, and can thus inform focused service delivery targeted at the specific needs of RD patients, pharmacoeconomic evaluation of orphan drugs, appropriate health and social service commissioning, and facilitation of clinical trials. It is also essential for current orphan drug legislation objectives to stimulate the development of RD treatments by incentivizing to compensate for the small market size. Definitions to-date however have not been based on robust evidence, as methodologically thorough analyses have not been possible due to insufficient epidemiological data, lack of scientific publications, and an absence of structured databases.

Estimating the global point prevalence of RDs is challenging foremost for the diversity of the data, which are derived from a variety of disparate information sources that are not standardized or that are difficult to combine, including published case reports or systematic reviews, patient registries, expert opinions, and other anecdotal evidence. This is aggravated by differing methods employed during case ascertainment and a lack of firmly established, and/or specific diagnostic criteria or coding systems to capture this data [15]. The nature of RDs raises further challenges because of the small number of cases, compounded by significant clinical heterogeneity. Some RDs vary in frequency across geographic area, due to population genetic diversity, environmental or societal pressures, or survival issues in different regions [16, 17]. Point prevalence of rare clinical presentations can be over- or underestimated due to their overlap with common comorbidities [18, 19]. Rapidly advancing genetic technologies identify new disease genes, thereby resulting in an initial increase in the number of known RDs. However, these same technologies ultimately decrease the number of RD classifications by ascribing unifying genetic diagnoses to disparate phenotypes.

Orphanet (www.orpha.net) is a 37-country network, cofunded by the European Commission that aims to increase knowledge on RDs so as to improve the diagnosis, care, and treatment of people with RDs [20–22]. The database is a comprehensive, manually curated and expert-reviewed knowledge-base specific for RDs, and is an IRDiRC Recognized Resource and Elixir Core Data Resource [23, 24]. Orphanet catalogues RDs: encompassing diseases, malformation syndromes, morphological, and biological anomalies, as well as particular clinical situations considered as ‘rare in Europe’. The catalogue is structured to provide a hierarchical representation classified by medical domain [25, 26], annotated with medical classifications, age of onset, inheritance, genes, and a directory of health and research resources.

**Fig. 1:**
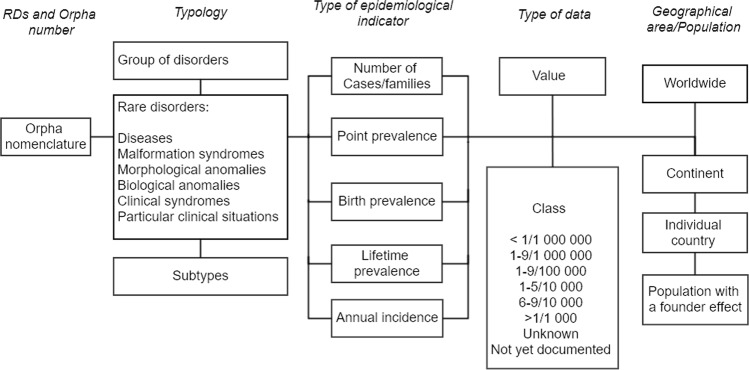
Representation of epidemiological data in the Orphanet database

Since 2005 Orphanet has annotated RD information with epidemiological indicators via a systematic data collection procedure [27], with 81.2% of RDs annotated by 2018. The methodology is an ongoing systematic literature survey of peer reviewed journals, specialized reports, registries, and international databases, with expert advice sought for epidemiological indicators not documented in the literature. The type of epidemiological indicator is recorded (point prevalence, annual incidence, birth prevalence, lifetime prevalence, case report, or family report) for each disease and are annotated with epidemiological data as numerical estimates, preestablished ranges (<1/1 000 000, 1–9/1 000 000, 1–9/100 000, 1–5/10 000, 6–9/10 000, and >1/1 000), or “not yet documented” and “unknown” (where despite intensive bibliographical research, no data could be found) (see Fig. 1). All data are annotated with a geographical area (country, continent, or worldwide) and/or a particular population if relevant (e.g., ethnic founder populations).

As RDs become a global public health priority [28] and a global national policy priority, it is essential to provide an evidence-based estimate of the prevalence of RDs. This article will analyse epidemiological data available in the reference database Orphanet to perform the first robust evaluation of the cumulative point prevalence of RDs.

## Methods

The ‘Orphanet Epidemiological file’ was obtained on October 1, 2018 [29]. Data for unique clinical rare diseases (defined as ‘disorders’ in the Orphanet classification) were included for analysis, excluding ‘groups of disorders’ (e.g., lysosomal diseases) and ‘disorders subtypes’ to avoid duplicate counts. Univariate analysis was performed on ‘age of onset’. ‘Genetic disorders’ were found in the ‘classification of rare genetic diseases’ and as such are defined as ‘known or suspected to be familial; inherited or de novo autosomal or x-linked single gene disorders; mitochondrial disorders; and chromosomal rearrangements’. The inheritance pattern was derived by analysis of disorders within the classifications ‘rare genetic diseases’ including ‘rare chromosomal anomalies’ and the annotations on ‘mode of inheritance’.

**Fig. 2:**
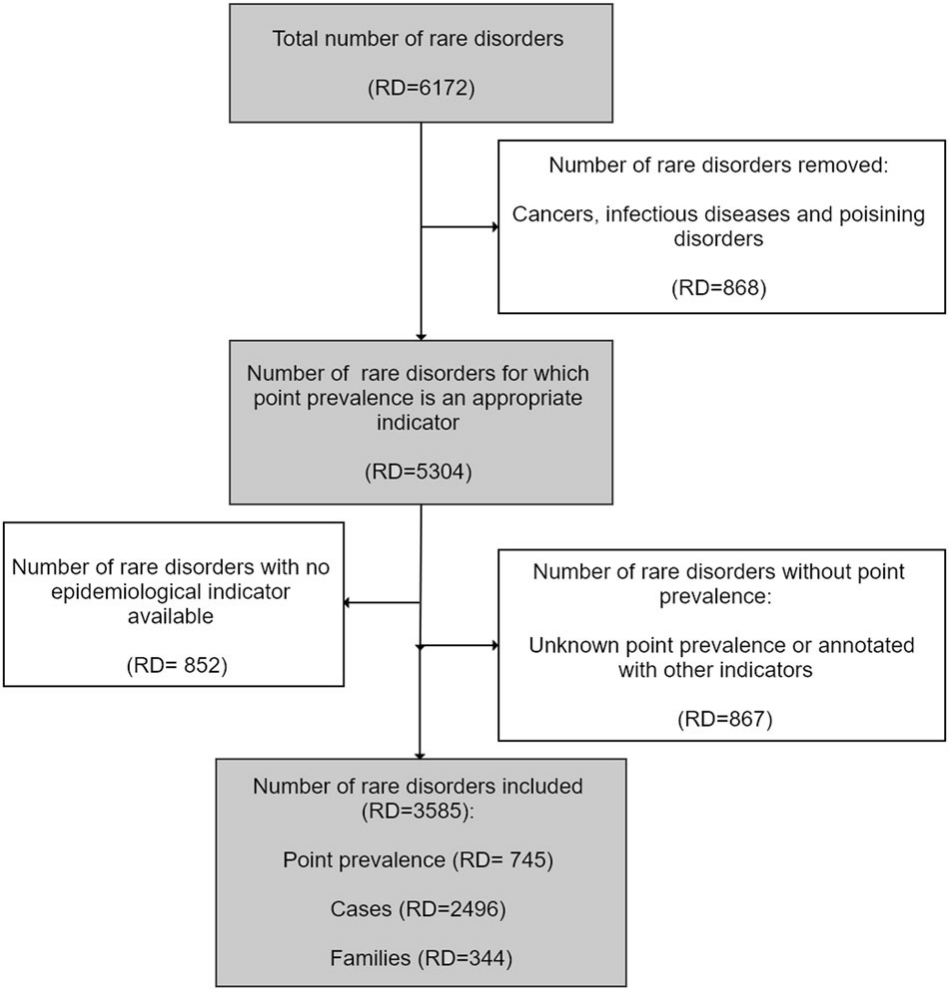
Selection process of point prevalence data from the Orphanet database’s epidemiological file for analysis

‘Point prevalence’, defined as the number of all the existing cases in a population at a specific point in time, was chosen as the epidemiological indicator for analysis. Epidemiological data in Orphanet is recorded as numerical values or as predefined ranges when numerical values are not available. A subset of data was selected for homogeneity from the heterogeneous epidemiological data as follows, as described in Fig. 2:

1. Data for groups of disorders and disorder subtypes were excluded.
2. Rare cancers, infectious diseases, and poisonings with an acute or subacute clinical course were excluded from analysis as they are described by ‘Incidence’ as the epidemiological indicator. Disorders were also excluded when point prevalence could not be calculated, such as those described by ‘prevalence at birth’, ‘lifetime prevalence’, or ‘annual incidence’.
3. Only one point prevalence per disorder was included; for disorders with more than one recorded geographic point prevalence, one value was selected in the order of preference: worldwide; or European (EU, Russia, Turkey, and Iceland). If no point prevalence was available from these regions then a USA point prevalence figure was used if it did not exceed the European threshold definition of 5/10 000. RDs that did not have a point prevalence reported from one of these geographic areas were excluded.
4. Disorders with a mean prevalence exceeding the threshold of 5/10 000 were excluded as they are not considered to be rare in Europe.

Data were of three types and were treated separately depending on whether the prevalence was (1) a numerical value (2) a class (predefined range), or (3) based on case and family reports.

### RDs with a numerical prevalence value

Mean prevalence values were used and assigned classes with the intervals (<1/1 000 000, 1–9/1 000 000, 1–9/100 000, and 1–5/10 000) to correspond with the prevalence categories listed on Orphanet.

### RDs with a prevalence range only

Data were verified to ensure that the prevalence range assigned concurred with the prevalence categories listed on Orphanet for comparability. No numerical median or mean value could be assigned within the prevalence range as the distribution of point prevalence data within each class was not known. Accordingly, for each RD, data were recorded as (i) the minimum value within each class was assigned (e.g., 1/100 000 for the class 1–9/100 000) and (ii) the maximum value within each class was assigned (e.g., 5/10 000 for the class 1–5/10 000) for each disease. For the class <1/1 000 000, both the minimum and maximum values were assigned as 1/1 000 000.

### Case and family reports

The point prevalence class was assumed to be <1/1 000 000 for each RD represented by only single cases or families. The point prevalence value was not calculated for each RD described by only case- and family- reports. Instead, indirect point prevalence was calculated for these RDs as a group as the point prevalence of the sum of all the case- and family- reports, divided by the global population in 2017 [30]. To ensure that changing family size has a negligible effect, we repeated our analysis using ten cases per family instead of one case per family.

$$Indirect\;po{\it{int}}\;prevalence = \frac{{\sum \;of\;cases\;and\;families\;reported}}{{Global\;population\;size}}$$

### Calculation of overall point prevalence estimates

Minimum and maximum boundaries of the global point prevalence estimate were calculated by summing the results of all three groups: all disorder-specific point prevalence values (for disorders with a point prevalence value), disorder-specific minimum values (for minimum boundary) or maximum values (for maximum boundary)(for disorders with point prevalence class only), and the indirect point prevalence estimate (derived for cases and families).

Point prevalence estimates were summarized descriptively and presented as the number of cases per 100 000. Step by step analyses of prevalence data and disease classifications were carried out using R-3.5.3 and Perl scripts, as described in the Supplemental Files.

## Results

**Fig. 3:**
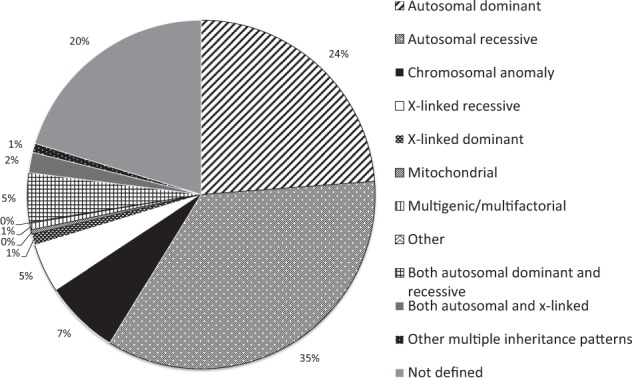
Distribution of inheritance patterns of genetic rare diseases. Genetic diseases were those in the ‘Orphanet classification of genetic diseases’, at the clinical entity ‘disorder’ level (excluding disorder groups and disorder subtypes)

Orphanet contains descriptions of 6172 clinically unique RDs excluding groups of disorders and disorders subtypes. Age of onset is described in 81.3% (*n* = 5018): 3510 (69.9%) are exclusively pediatric onset; 908 (18.2%) have onset spanning both pediatric and adult groups and 600 (11.9%) are exclusively adult onset. 4440 RDs (71.9%) are classified as genetic. Of the genetic RDs, 79.7% were annotated with one (72.4%) or more (7.3%) inheritance patterns, as described in Fig. 3 and Supplemental Table 1.

Of the 6172 unique RDs (Fig. 2), 5304 RDs remained once disease described by incidence (cancers: 11.1%, infectious diseases: 2.6%, and poisonings: 0.4%) were excluded (*n* = 868, 14.1%). Data were excluded for 1719 RDs: those annotated with epidemiological figures other than point prevalence; those with ‘unknown’ point prevalence; or those not yet documented. A total of 3585 RDs with point prevalence data were included in this analysis, representing 67.6% of the RDs for which point prevalence is the pertinent epidemiological indicator: 745 RDs annotated with a point prevalence figure or class (20.8%), 2496 RDs described by reports of single cases (69.6%), and 344 described by reports of families (9.6%).

**Fig. 4:**
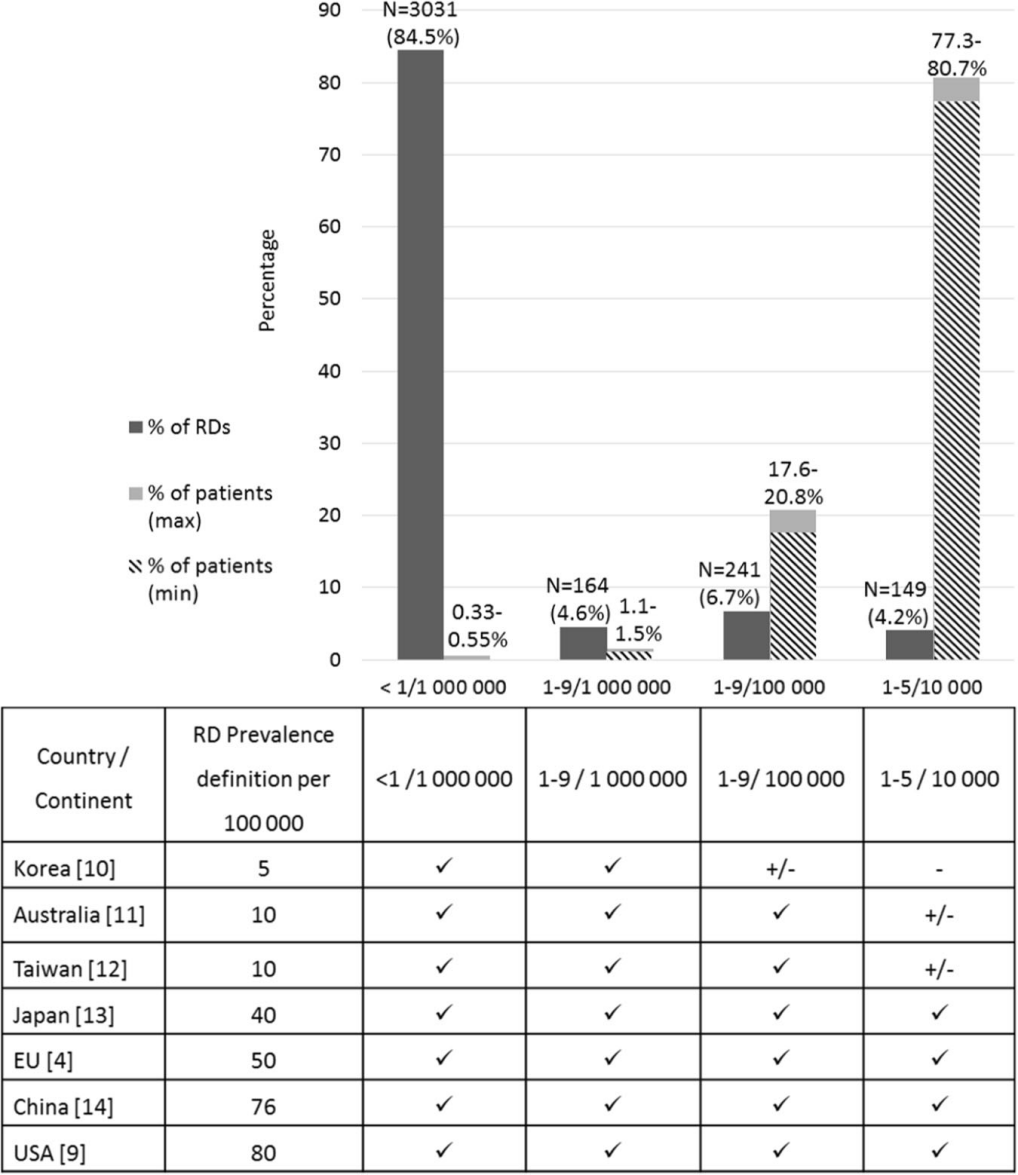
Distribution of rare diseases and rare disease patients according to the point prevalence class. For each prevalence class both the number of rare diseases and the range of patients with rare diseases are shown. The inclusivity of each prevalence class in national definitions of ‘rare disease’ is shown below

Figure 4 shows the distribution of the RDs included in the analysis according to their point prevalence class.

### Global point prevalence

Table 1 shows that the total number of cases and families were 85 680 and 3418, respectively. When extrapolated to the worldwide population in 2017 (7 550 000 000 people) [20] the indirect estimated point prevalence was 1.2 per 100 000. When the analysis was repeated using ten cases per family (10 times the previous estimate), the estimate remained relatively stable at 1.6 per 100 000—due to the small value of the numerator compared with the denominator. The indirect point prevalence of 1.2 per 100 000 was used in further calculations.

**Table 1:** Derivation of global point prevalence from case reports; family reports; preestablished ranges and numerical values. **a** Total number of cases divided by the worldwide population (7 550 000 000); **b** indirect point prevalence increases 1.6 if number of cases = number of families × 10; 1.2 used for all further calculations; **c** minimum and maximum boundaries

Indicators	Rare diseases		Number of cases	Point prevalence estimates per 100 000
	Number	%		
Cases/families reports				
Cases	2496	69.6	85 680	–
Families	344	9.6	3 418	–
Prevalence				
Indirect point prevalence (a)	2840	79.2	89 098	1.2 (b)
Point prevalence	745	20.8	–	3 481.1–5 909.1 (c)
Total (sum)	3585	100	–	3 482.3–5 910.3

When this indirect point prevalence (Table 1) was summed with the prevalence from the epidemiological data registered for the selected RDs, the estimated minimum and maximum boundaries for global point prevalence of RDs was calculated as 3 482.3–5 910.3 per 100 000 (~3.5–5.9%) in the general population. This forecasts 17.8–30.3 million people in the European Union and 262.9–446.2 million people worldwide are affected by a RD (2017 population [30, 31]).

The entire analysis was repeated, stratified to obtain minimum and maximum boundaries for cumulative point prevalence for each prevalence class (Fig. 4). More than 98% of the people with RDs were found among the 390 diseases in the categories (1–9/100 000) and (1–5/10 000).

## Discussion

While research in RDs has proved to be helpful to elucidate the mechanism and the natural history of individual diseases or groups of diseases, epidemiological data on the overall burden of RDs, a disparate group of diseases with a wide biological diversity, are anecdotal. This study challenges the estimate that RDs affect 6–8% of the global population [8]. Most RDs (85.9%) should be described by prevalence as an epidemiological indicator. Through our analysis, we estimate that the global point prevalence of RDs is at least 3.5–5.9%, derived from point prevalence estimates for 67.6% of RDs for which prevalence is a pertinent epidemiological indicator. True cumulative point prevalence for all RDs is likely to be higher; however, we cannot reasonably infer an estimate from our data to all RDs.

Our study offers a more global, evidence based, and comprehensive approach to estimating the prevalence of RDs than previous studies. The often quoted ‘rare diseases affect 6–8% of the population’ [8] was first used in Europe in 1992–3 French research documents that became the basis of the EU Legislation on Orphan Products in 1999 [4]. An author of these documents cites that the figure was drawn from American National Organization for Rare Disease and National Institutes of Health Office of Rare Diseases publications in the 1990s (Y Le Cam, personal communication). However, the 2010 US National Academy report on Rare Diseases and Orphan Products [32] states that in the original 1989 National Commission on Orphan Diseases publication [33] “… estimates were not accompanied by analysis or substantive citation of sources”.

The Veneto regional registry [34] estimates the cumulative RD prevalence of 3.3 per 1000, based on information from 58% of RDs present in Orphanet (2012). RD prevalence was estimated as 2.0% in Western Australia [35] and 1.5% in Hong Kong [36] through hospital inpatient data collected on 467 diseases, capturing data from only 13% of the RDs in our study. Compared with these estimates, our study has a larger disease base, and a wider population coverage.

Ferreira’s [37] use of data from the Orphanet Report Series on Epidemiological data [38] to calculate a cumulative prevalence of 6.2% of the general population by considering 798 RDs has limitations as this file contains only a subset of data from Orphadata. The report series file lacks the structured epidemiological data of Orphadata and thus contains the inclusion of overlapping data by including groups, disorders, and subtypes (e.g., Hemophilia B and mild hemophilia B) that are not independent, which would lead to prevalence overestimates. The report series data contains only worldwide and European data. Ferreira’s study also does not include prevalence figures derived from case reports and families, which are the majority of rare diseases. Our analysis limits itself to unique clinical disorders, so that there are no double counts by inclusion of groups or subtypes.

Our estimate of genetic diseases of 71.9% differs from the 39% derived by Ferreira [37] for two reasons— Ferreira defines ‘genetic’ diseases as those with a single gene annotating a disease entry (derived from the ‘Orphadata: Genes’ file); and overestimates the total number of disease as groups and subtypes have been included as well as unique clinical entities. We have used a broader, more practical definition of ‘genetic’, including not only known single genes but diseases known or suspected to be familial with no underlying gene identified; mitochondrial diseases; and chromosomal rearrangements; as derived from the ‘Orphanet classification of genetic disorders’ by methods described in our supplemental files; and we have excluded double-counts by considering only the number of unique clinical entities.

Although the 14.1% of RDs described by incidence could not be considered in this analysis, the contribution of RDs measured by annual incidence, such as rare cancers, should be taken into account. For example, analyses of the RARECARE European population-based rare cancer registry data estimated that 4 300 000 people were living in the European Union with a diagnosis of a rare cancer [39].

Our approach to determining overall RD point prevalence also has several potential limitations. The identification of the high-quality population-based studies is difficult in the context of RDs, despite existing guidelines [40]. Collection of data is hampered by heterogeneity in the epidemiological approaches used to estimate the point prevalence, inconsistency in reporting ‘incidence’ and ‘prevalence’, and the use of anecdotal data.

Orphanet, as a European based resource, registers RDs with a point prevalence of <5 per 10 000, and diseases defined as rare in most jurisdictions will be included in Orphanet’s epidemiological review. Countries with a more restrictive ‘rare disease’ definition may not collect or publish data on diseases considered rare in Europe. Conversely, Orphanet excludes diseases considered to be rare in countries with more permissive definitions. Orphanet’s reliance on publications in peer-reviewed journals may exclude relevant data published elsewhere.

The inclusion of only worldwide, European or American point prevalence values in our study could have a significant effect on our estimate of global point prevalence—either to underestimate the contribution of RDs that are not prevalent in Europe or the US but are prevalent elsewhere, or to overestimate the generalizability of European and American RDs to the rest of the world. The decision to select geographic regions was based on factors including the lack of RD epidemiological information from many areas of the world such as India, China, South America, and Africa; and the publication bias that results in mostly European and North American studies being published in the literature reviewed in Orphanet data collection. However, as incident diseases were excluded from the analysis, some bias is likely limited by the exclusion of infectious diseases.

Geographic or population variability is recognised in many RDs, and is relevant among the almost 72% of RDs that we have shown to have a genetic basis. Our model that one-point prevalence can represent a RD is an overgeneralization. In addition, the complex nature of RDs may lead to variation in epidemiological estimates, for example by the lack of diagnostic consensus for many RDs [41]. Late onset, reduced penetrance, under-recognition, and variable presentations of some RDs, as well as the lack of population screening tools for many RDs, can affect the recorded point prevalence [42].

Circumventing these limitations to avoid the ad hoc and biased nature of scientific publications and to provide an accurate point prevalence estimate requires systematic capture of data from health information systems. Underrepresentation of RDs in coding systems necessitates use of a specific codification system such as the Orphanet nomenclature of RD (ORPHAcodes) [43]. RD registration would not only yield prevalence data but could also provide data on: the burden of disease, life expectancy, medical system use, and health service benchmarking. Registries should be population-based across all RDs, to allow country-specific data collection and to demonstrate the population variability inherent in RDs [44]. Initial examples of RD registration show that population point prevalence can be derived [34] and burden of RDs on a population can be calculated [35, 36]. Until such data are more widely available however, our study offers the best possible estimation of the global cumulative point prevalence of RDs.

Our research highlights disparities in even the definition of ‘rare disease’. Although published national definitions of the point prevalence of RDs range from 5 to 80 per 100 000 population (Fig. 4), the distribution of the point prevalence of individual diseases in the Orphanet database shows that even the lowest point prevalence definition of 5 per 100 000 encompasses at least 90.5 % of the RDs recorded on Orphanet, with the great majority (84.5%) derived from very low point prevalence diseases (<1/1 000 000). However, an examination of the percentage of patients attributable to each prevalence category differs: the exclusion of the (1–5/10 000) category in the Korean, Australian, and Taiwanese RD definitions excludes ~80% of the patients that would be considered to have RDs in Europe. Definitions based on absolute number of people with RDs, as in the USA, result in decreasing point prevalence figures over time as the population grows: in 1983, the US point prevalence corresponded to 8.6/10 000, whereas in 2017 it was 6.1/10 000; a figure close to the EU definition. Defining RD frequency by point prevalence allows resource planning with population growth. The recognition of a common definition for RDs would promote research cooperation and information sharing in an increasingly globalized approach to research and care and favours the adoption of a common international framework for RDs. Furthermore, future research defining the distribution of RD by medical domain according to prevalence would provide evidence for designing regional, national, and global strategies. These would directly benefit the vast majority of patients with the most prevalent RDs and create the necessary framework of expertise to serve patients with the rarest such diseases.

In conclusion, refinement of the epidemiological estimate of RDs is timely as RDs become a global policy priority of ‘leaving no one behind’ [45] and the United Nations, World Health Organization, Organization for Economic Co-operation and Development, Asia-Pacific Economic Cooperation as well as countries move toward adoption of RD policies and programs. We found that RDs affect at least 3.5–5.9% of the worldwide population. This point prevalence translates into conservative figures of 18–30 million persons in the EU, and 263–446 million persons affected worldwide by RDs at any point in time. As this analysis did not consider rare cancers, infectious diseases and poisonings, the number of people affected by RDs is likely considerably higher. Further research, notably through long-term population registries and the implementation of a specific codification for the identification of RD patients in healthcare systems, will help to refine the estimates.

## Supplementary information

Supplemental Figure 1

Supplemental Figure 2

Supplemental table 1

Supplemental Material 1

Supplemental Material 2

## Data Availability

Orphadata [http://www.orphadata.org. XML data version 1.2.4/4.1.6 [2016–06–01] (orientdb version), accessed October 1st, 2018].
