# Supplementary material for: Estimating cumulative point prevalence of rare diseases: analysis of the Orphanet database
Source: Eur J Hum Genet. 2019 Sep 16;28(2):165–73. doi: 10.1038/s41431-019-0508-0 (PMC6974615; doi:10.1038/s41431-019-0508-0)
Supplement: Supplementary file 1 — Supplemental Figure 1 [file 41431_2019_508_MOESM1_ESM.pptx]

## Slide 1
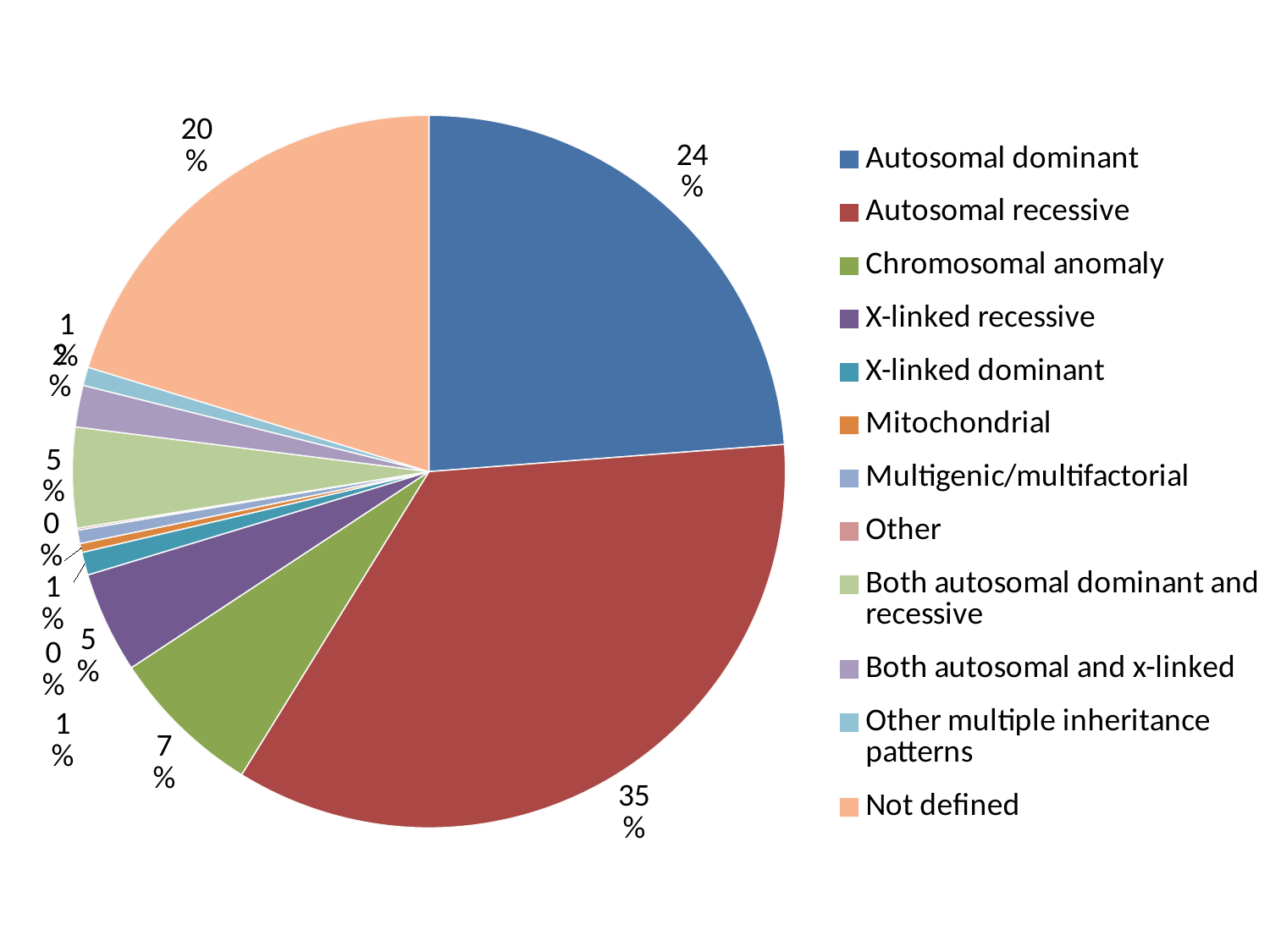

### Chart
| Category | % |
|---|---|
| Autosomal dominant | 0.23783783783783785 |
| Autosomal recessive | 0.3502252252252252 |
| Chromosomal anomaly | 0.06914414414414415 |
| X-linked recessive | 0.04594594594594595 |
| X-linked dominant | 0.01036036036036036 |
| Mitochondrial | 0.004054054054054054 |
| Multigenic/multifactorial | 0.006081081081081081 |
| Other | 0.0009009009009009009 |
| Both autosomal dominant and recessive | 0.045495495495495496 |
| Both autosomal and x-linked | 0.01891891891891892 |
| Other multiple inheritance patterns | 0.008333333333333333 |
| Not defined | 0.20270270270270271 |
