# Supplementary figures and images for: Estimating cumulative point prevalence of rare diseases: analysis of the Orphanet database
Source: Eur J Hum Genet. 2019 Sep 16;28(2):165–73. doi: 10.1038/s41431-019-0508-0 (PMC6974615; doi:10.1038/s41431-019-0508-0)

## Slide 1
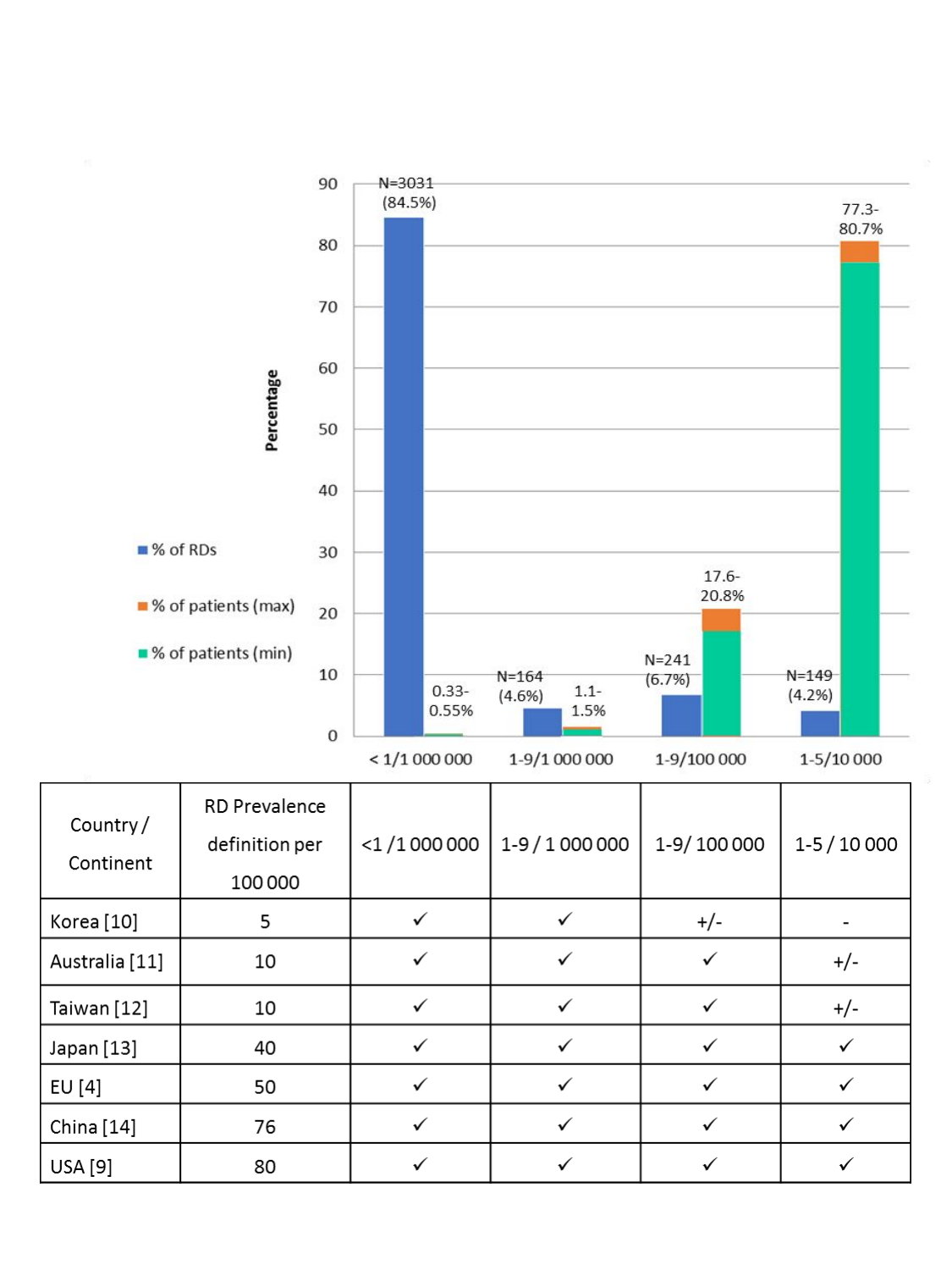

Supplement: Supplementary file 2 — Supplemental Figure 2 [file 41431_2019_508_MOESM2_ESM.pptx]
