# Supplementary material for: Estimating cumulative point prevalence of rare diseases: analysis of the Orphanet database
Source: Eur J Hum Genet. 2019 Sep 16;28(2):165–73. doi: 10.1038/s41431-019-0508-0 (PMC6974615; doi:10.1038/s41431-019-0508-0)
Supplement: Supplementary file 3 — Supplemental table 1 [file 41431_2019_508_MOESM3_ESM.docx]

| **Inheritance grouping** | **N for inheritance group** | **N for inheritance pattern** | **% of Genetic Rare diseases** |
| --- | --- | --- | --- |
| ***Sub-classification by inheritance pattern*** |  |  |  |
| Autosomal dominant | 1056 |  | 23,78 |
| Autosomal recessive | 1555 |  | 35,02 |
| Chromosomal anomaly | 307 |  | 6,91 |
| X-linked recessive | 204 |  | 4,59 |
| X-linked dominant | 46 |  | 1,04 |
| Mitochondrial | 18 |  | 0,41 |
| Multigenic/multifactorial | 27 |  | 0,61 |
| Other | 4 |  | 0,09 |
| *Semi-dominant* |  | 3 |  |
| *Y-linked* |  | 1 |  |
| Both autosomal dominant and recessive | 202 |  | 4,55 |
| Both autosomal and X-linked | 84 |  | 1,89 |
| *Autosomal dominant;Autosomal recessive;X-linked recessive* |  | 31 |  |
| *Autosomal recessive;X-linked recessive* |  | 23 |  |
| *Autosomal dominant;X-linked recessive* |  | 12 |  |
| *Autosomal dominant;X-linked dominant* |  | 8 |  |
| *Autosomal dominant;Autosomal recessive;X-linked dominant* |  | 4 |  |
| *Autosomal recessive;X-linked dominant* |  | 3 |  |
| *Autosomal dominant;Autosomal recessive;X-linked recessive;Y-linked* |  | 2 |  |
| *Autosomal dominant;Autosomal recessive;X-linked recessive;X-linked dominant* |  | 1 |  |
| Other multiple inheritance patterns | 37 |  | 0,83 |
| Autosomal dominant;Multigenic/multifactorial |  | 9 |  |
| X-linked recessive;X-linked dominant |  | 6 |  |
| Autosomal recessive;Mitochondrial inheritance |  | 5 |  |
| Autosomal dominant;Autosomal recessive;Multigenic/multifactorial |  | 4 |  |
| Autosomal dominant;Autosomal recessive;X-linked recessive;Mitochondrial inheritance |  | 3 |  |
| Autosomal dominant;Mitochondrial inheritance |  | 3 |  |
| Autosomal dominant;Autosomal recessive;Mitochondrial inheritance |  | 2 |  |
| Autosomal dominant;Autosomal recessive;Multigenic/multifactorial;X-linked recessive |  | 1 |  |
| Autosomal recessive;Mitochondrial inheritance;X-linked dominant |  | 1 |  |
| Autosomal recessive;Multigenic/multifactorial |  | 1 |  |
| Autosomal recessive;Oligogenic |  | 1 |  |
| X-linked recessive;Mitochondrial inheritance |  | 1 |  |
| Not defined | 900 |  | 20,27 |
| *Absent* |  | 654 |  |
| *Unknown* |  | 140 |  |
| *Not applicable* |  | 94 |  |
| *No data available* |  | 12 |  |
| **Total** | 4440 | 1025 | 100,00 |
