# Supplementary material for: Estimating cumulative point prevalence of rare diseases: analysis of the Orphanet database
Source: Eur J Hum Genet. 2019 Sep 16;28(2):165–73. doi: 10.1038/s41431-019-0508-0 (PMC6974615; doi:10.1038/s41431-019-0508-0)
Supplement: Supplementary file 4 — Supplemental Material 1 [file 41431_2019_508_MOESM4_ESM.docx]

**Titles and legends to supplemental figures**

Supplemental Figure 1: Color version of Figure 3: Distribution of inheritance patterns of genetic rare diseases. Genetic diseases were those in the ‘Orphanet Classification of Genetic Diseases’.

Supplemental Figure 2: Color version of Figure 4: Distribution of rare diseases and rare disease patients according to the point prevalence class. For each prevalence class both the number of rare diseases and the range of patients with rare diseases are shown. The inclusivity of each prevalence class in national definitions of ‘rare disease’ is shown below.

**Titles and legends to supplemental tables**

Supplemental table 1: Data supporting Figure 3 and Supplemental Figure 1: Complete distribution of inheritance patterns of genetic rare diseases from the ‘Orphanet Classification of Rare Diseases’, at the clinical entity ‘disorder’ level (excluding disorder groups and disorder sub-types).
