# Supplementary material for: Estimating cumulative point prevalence of rare diseases: analysis of the Orphanet database
Source: Eur J Hum Genet. 2019 Sep 16;28(2):165–73. doi: 10.1038/s41431-019-0508-0 (PMC6974615; doi:10.1038/s41431-019-0508-0)
Supplement: Supplementary file 5 — Supplemental Material 2 [file 41431_2019_508_MOESM5_ESM.docx]

##################################################################

## **Estimating cumulative point prevalence of rare diseases: analysis of the Orphanet database** ##

##################################################################

# Written by Stephanie Nguengang Wakap and Daniel Murphy. If you have any questions or comments, contact us over email [stephanie.nguengang-wakap@inserm.fr](mailto:stephanie.nguengang-wakap@inserm.fr) , [DanielMurphy@mater.ie](mailto:DanielMurphy@mater.ie) , [annie.olry@inserm.fr](mailto:annie.olry@inserm.fr).

**# (Our results could likely be different from those that will be generated as Orphadata files are updated on a regular basis)**

**######################**

**## Determination of the total number of rare disorders ##**

**######################**

# Go to orphadata.org, epidemiology directory, download file <http://www.orphadata.org/data/xml/en_product9_prv.xml> to desktop and save as xml file.  Open this xml in Excel and then save as a csv file ‘Rare_diseases_Epidemiology.csv’

######################

## Import data ##

######################

#To import data, follow the instructions below. This will import the orphanet rare diseases epidemiological data file assuming that the file is saved as a csv file in your working directory using the name "epi" and that the first row contains variable names.

epi<-read.csv("Rare_diseases_Epidemiology.csv",header=TRUE,sep=";",stringsAsFactors=FALSE,na.strings="")

#Keep relevant variables

rd1<-epi[, c("OrphaNumber","Name","Name6")]

#Rename variable ’Name6’ with 'DisorderType'

rd1$DisorderType<-rd1$Name6

# Exclude groups of disorders and disorders sub-types

disorders0<-rd1[rd1$DisorderType %in% c("Biological anomaly", "Clinical syndrome","Disease","Malformation syndrome","Morphological anomaly","Particular clinical situation in a disease or syndrome"),]

#Remove duplicates

disorders<-disorders0[!duplicated(disorders0$OrphaNumber),]

**#Total number of rare disorders**

nrow(disorders)

**######################**

**## Distributions of rare disorders by age of onset ##**

**######################**

# Go to orphadata.org, epidemiology directory, download file <http://www.orphadata.org/data/xml/en_product9_ages.xml> to desktop and save as xml file.  Open this xml in Excel, and then save as a csv file ‘Rare_diseases_Natural_history.csv’

######################

## Import data ##

######################

rdnh<-read.csv("Rare_diseases_Natural_history.csv",header=TRUE,sep=";",stringsAsFactors=FALSE,na.strings="")

#Keep relevant variables

rdnh1<-rdnh[,c("OrphaNumber","Name","Name11")]

#Rename variable ‘Name11’ with ‘Onset’

rdnh1$Onset<-rdnh1$Name11

# Merge Natural history table with the rare disorders table

rdonset<-merge(disorders,rdnh1,by="OrphaNumber",all.x=T)

# Exclude rare disorders without information on age of onset

rdonset1<-rdonset[which(!is.na(rdonset$Onset)),]

# Exclude rare disorders for which no data is available

rdonset2<-rdonset1[which(rdonset1$Onset !="No data available"),]

# Duplicate suppression (this gives the number of rare disorders with an age of onset)

rdonset3<-rdonset2[!duplicated(rdonset2$OrphaNumber),]

nrow(rdonset3)

# List of pediatric rare disorders

pedia<-rdonset2[rdonset2$Onset%in% c("Antenatal","Neonatal","Infancy","Childhood","Adolescent","All ages"),]

pedia1<-pedia[!duplicated(pedia$OrphaNumber),]

nrow(pedia1)

#Proportion of pediatric rare disorders

nrow(pedia1)/nrow(rdonset3)*100

# List of adult onset rare disorders

adult0<-rdonset2[rdonset2$Onset %in% c("Adult","Elderly","All ages"),]

adult1<-adult0[!duplicated(adult0$OrphaNumber),]

nrow(adult1)

nrow(adult1)/nrow(rdonset3)*100

adult2<-adult1[,c("OrphaNumber","Name.x","DisorderType","Onset")]

#Merge with the table of pediatric rare disorders

pedia_adult<-merge(pedia1,adult2,by="OrphaNumber",all.x=T)

**### Rare disorders which are exclusively pediatric**

pedia_exclusiv<-pedia_adult[which(is.na(pedia_adult$Onset.y)),]

nrow(pedia_exclusiv)

**#Proportion of rare disorders which are exclusively pediatric**

nrow(pedia_exclusiv)/nrow(rdonset3)*100

**### Rare disorders which are exclusively adult onset**

adult_pedia<-merge(adult2,pedia1,by="OrphaNumber",all.x=T)

adult_exclusiv<-adult_pedia[which(is.na(adult_pedia$Onset.y)),]

nrow(adult_exclusiv)

**#Proportion of rare disorders which are exclusively adult onset**

nrow(adult_exclusiv)/nrow(rdonset3)*100

**# Number of Rare disorders with both pediatric and adult onset**

all<-nrow(rdonset3)-(nrow(pedia_exclusiv)+nrow(adult_exclusiv))

all/nrow(rdonset3)*100

**######################**

**## Determination of the proportion of rare genetic disorders ##**

**######################**

# Go to orphadata.org, rare disease/Classification directory, download file <http://www.orphadata.org/data/xml/en_product3_156.xml> to desktop and save as xml file.  Open this xml in Excel, and then save as a csv file ‘Rare_genetic_diseases.csv’

######################

## Import data ##

######################

rgd<-read.csv("Rare_genetic_diseases.csv",header=TRUE,sep=";",stringsAsFactors=FALSE,na.strings="")

#Create the list of rare genetic diseases

# Step 1: use of Orphanumbers and Name of diseases

rgd1<-rgd[,c("OrphaNumber","Name")]

rgd2<-rgd[,c("OrphaNumber7","Name10")]

rgd3<-rgd[,c("OrphaNumber14","Name17")]

rgd4<-rgd[,c("OrphaNumber21","Name24")]

rgd5<-rgd[,c("OrphaNumber28","Name31")]

rgd6<-rgd[,c("OrphaNumber35","Name38")]

rgd7<-rgd[,c("OrphaNumber42","Name45")]

rgd8<-rgd[,c("OrphaNumber49","Name52")]

rgd9<-rgd[,c("OrphaNumber56","Name59")]

rgd10<-rgd[,c("OrphaNumber63","Name66")]

rgd11<-rgd[,c("OrphaNumber70","Name73")]

#remove duplicate entities

rgd1s<- rgd1 [!duplicated(rgd1$OrphaNumber),]

rgd2s<- rgd2 [!duplicated(rgd2$OrphaNumber7),]

rgd3s<- rgd3 [!duplicated(rgd3$OrphaNumber14),]

rgd4s<- rgd4 [!duplicated(rgd4$OrphaNumber21),]

rgd5s<- rgd5 [!duplicated(rgd5$OrphaNumber28),]

rgd6s<- rgd6 [!duplicated(rgd6$OrphaNumber35),]

rgd7s<- rgd7 [!duplicated(rgd7$OrphaNumber42),]

rgd8s<- rgd8 [!duplicated(rgd8$OrphaNumber49),]

rgd9s<- rgd9 [!duplicated(rgd9$OrphaNumber56),]

rgd10s<- rgd10 [!duplicated(rgd10$OrphaNumber63),]

rgd11s<- rgd11 [!duplicated(rgd11$OrphaNumber70),]

# rename variables

rgd2s $ OrphaNumber <- rgd2s $ OrphaNumber7

rgd3s $ OrphaNumber <- rgd3s $ OrphaNumber14

rgd4s $ OrphaNumber <- rgd4s $ OrphaNumber21

rgd5s $ OrphaNumber <- rgd5s $ OrphaNumber28

rgd6s $ OrphaNumber <- rgd6s $ OrphaNumber35

rgd7s $ OrphaNumber <- rgd7s $ OrphaNumber42

rgd8s $ OrphaNumber <- rgd8s $ OrphaNumber49

rgd9s $ OrphaNumber <- rgd9s $ OrphaNumber56

rgd10s $ OrphaNumber <- rgd10s $ OrphaNumber63

rgd11s $ OrphaNumber <- rgd11s $ OrphaNumber70

rgd2s $ Name <- rgd2s $ Name10

rgd3s $ Name <- rgd3s $ Name17

rgd4s $ Name <- rgd4s $ Name24

rgd5s $ Name <- rgd5s $ Name31

rgd6s $ Name <- rgd6s $ Name38

rgd7s $ Name <- rgd7s $ Name45

rgd8s $ Name <- rgd8s $ Name52

rgd9s $ Name <- rgd9s $ Name59

rgd10s $ Name <- rgd10s $ Name66

rgd11s $ Name <- rgd11s $ Name73

#Keep relevant variables

rgd2ss<-rgd2s[,c("OrphaNumber","Name")]

rgd3ss<-rgd3s[,c("OrphaNumber","Name")]

rgd4ss<-rgd4s[,c("OrphaNumber","Name")]

rgd5ss<-rgd5s[,c("OrphaNumber","Name")]

rgd6ss<-rgd6s[,c("OrphaNumber","Name")]

rgd7ss<-rgd7s[,c("OrphaNumber","Name")]

rgd8ss<-rgd8s[,c("OrphaNumber","Name")]

rgd9ss<-rgd9s[,c("OrphaNumber","Name")]

rgd10ss<-rgd10s[,c("OrphaNumber","Name")]

rgd11ss<-rgd11s[,c("OrphaNumber","Name")]

# list of rare genetic diseases

genetic<-rbind(rgd1s,rgd2ss, rgd3ss, rgd4ss, rgd5ss, rgd6ss, rgd7ss, rgd8ss, rgd9ss, rgd10ss, rgd11ss)

genetic<-genetic [!duplicated(genetic$OrphaNumber),]

# Merge rare diseases table with the table of rare disorders

rd_gen<-merge(disorders,genetic,by="OrphaNumber",all.x=T)

# Keep rare genetic disorders

GENETICS<-rd_gen[which(!is.na(rd_gen$Name.y)),]

View(GENETICS)

#Total number of rare genetic disorders

nrow(GENETICS)

**#Calculate the proportion of rare genetic disorders**

nrow(GENETICS)/ nrow(disorders)*100

#Export the table of genetic disorders

write.table(GENETICS, file = " All_genetics.txt", sep = "\t",row.names = TRUE, col.names = NA)

**######################**

**## Distribution of rare genetic disorders ##**

**######################**

# Go to orphadata.org, epidemiology directory, download file <http://www.orphadata.org/data/xml/en_product9_ages.xml> to desktop and save as xml file.  Open this xml in Excel, and then save as a csv file ‘Rare_diseases_Natural_history.csv’

######################

## Import data ##

######################

rdh<-read.csv("Rare_diseases_Natural_history.csv",header=TRUE,sep=";",stringsAsFactors=FALSE,na.strings="")

#Keep relevant variables

rdh1<-rdh[,c("OrphaNumber","Name","Name21")]

#Rename variable ‘Name21’ with ‘Inheritance’

rdh1$Inheritance<-rdh1$Name21

# Merge the table of Inheritance with the table of rare disorders

rdinh<-merge(disorders, rdh1,by="OrphaNumber",all.x=T)

# Exclude rare disorders without information on inheritance

INHERITANCE <-rdinh[which(!is.na(rdinh$ Inheritance)),]

#Export the table of rare disorders with mode of inheritance

write.table(INHERITANCE, file = "All_inheritance.txt", sep = "\t",

row.names = TRUE, col.names =NA)

**######################**

**## PERL SCRIPT ##**

**######################**

#!/usr/bin/perl

use strict;

use warnings;

use XML::Simple;

use Data::Dumper;

use List::MoreUtils qw(uniq);

# create object

my $xml = new XML::Simple;

my $chrom_file = 'en_product3_147.xml';

my $rare_genetic_file = 'en_product1.xml';

my $disease_file = 'All_genetics.csv';

my $inheritance_file = 'All_inheritance.csv';

# to download files need the url

my $url = 'http://www.orphadata.org/data/xml/';

# download the xml files

dload($url, $chrom_file);

dload($url, $rare_genetic_file);

# read genetic disease file

my $genetic_data = $xml->XMLin($rare_genetic_file);

# create hash for orpha number

my %orpha_number;

my %genetic_disorders = %{ $genetic_data->{DisorderList}->{Disorder} };

for my $dis_key (keys %genetic_disorders) {

if ($genetic_disorders{$dis_key}->{DisorderType}->{Name}->{content} eq 'Disease' || $genetic_disorders{$dis_key}->{DisorderType}->{Name}->{content} eq 'Malformation syndrome' || $genetic_disorders{$dis_key}->{DisorderType}->{Name}->{content} eq 'Biological anomaly' || $genetic_disorders{$dis_key}->{DisorderType}->{Name}->{content} eq 'Clinical syndrome' || $genetic_disorders{$dis_key}->{DisorderType}->{Name}->{content} eq 'Malformation syndrome' || $genetic_disorders{$dis_key}->{DisorderType}->{Name}->{content} eq 'Morphological anomaly' || $genetic_disorders{$dis_key}->{DisorderType}->{Name}->{content} eq 'Particular clinical situation in a disease or syndrome') {

# use the pat id as key for the orpha, disease and type hashes

$orpha_number {$dis_key} = $genetic_disorders{$dis_key}->{OrphaNumber};

}

}

# create an inheritance hash

my %inheritance;

# read chromosomal anomaly XML file

my $chrom_data = $xml->XMLin($chrom_file);

my %anomaly_group = %{ $chrom_data->{DisorderList}->{Disorder}};

for my $anomaly (keys %anomaly_group) {

if ($anomaly eq 'ClassificationNodeList'){

my %chrom_classifications = %{ $anomaly_group{$anomaly}->{ClassificationNode}->{ClassificationNodeChildList}};

for my $class_key (keys %chrom_classifications) {

if ($class_key eq 'ClassificationNode'){

my @chrom_disorders = @{ $chrom_classifications{$class_key}};

foreach my $element (@chrom_disorders){

# the pat id for chromosomal anomalies is 10474

if ($element->{Disorder}->{id} eq 10474) {

my %chrom_disorders1 = %{$element->{ClassificationNodeChildList}};

# extract all cases where id is present (Pat id)

my $dumped = Dumper (\%chrom_disorders1);

my @dumped = split ("\n", $dumped);

@dumped = grep(/'id' => /, @dumped);

# make sure we're just left with the id

foreach my $id (@dumped) {

$id =~ s/\s//g;

$id =~ s/\'id\'=>\'//g;

$id =~ s/\'//g;

$id =~ s/,//g;

$inheritance {$orpha_number{$id}} = "Chromosomal anomaly";

}

}

}

}

}

}

}

# open the genetic disease file

open (DISFILE, $disease_file);

# create hash of genetic diseases with orpha number as key

my %disease;

my $dummy_dis = <DISFILE>; #First line is read here

while (my $line = <DISFILE>) {

my ($orpha_number, $name, $rest) = split (/;/, $line);

if (exists $disease {$orpha_number}) {

print "WARNING ", $disease {$orpha_number}, "is duplicated!\n";

} else {

$disease {$orpha_number} = $name;

}

}

close DISFILE;

# now open the inheritance file

open (INHFILE, $inheritance_file);

my $dummy_inh = <INHFILE>; #First line is read here

while (my $line = <INHFILE>) {

# separate at semi colons unless they're in brackets like with translocations

my ($orpha_number, $name, $type1, $type2, $name2, $inh1, $inheritance) = split (m/;(?![^()]*\))/, $line);

# check the inheritance patterns are the same

chomp $inheritance;

# remove special characters

$inheritance =~ s/[^[:print:]]//g;

# check last 2 columns are the same

if ($inh1 ne $inheritance){

print "Inheritance patterns differ: ", $orpha_number, ":", $inh1, ":", $inheritance, "\n";

}

# if the inheritance is already chromosomal anomaly then do nothing

if (exists $inheritance{$orpha_number}) {

if ($inheritance {$orpha_number} eq "Chromosomal anomaly") {

# do nothing

} elsif ($inheritance{$orpha_number} eq 'Unknown' || $inheritance{$orpha_number} eq 'Not applicable' || $inheritance{$orpha_number} eq 'No data available'){

$inheritance{$orpha_number} = $inheritance;

} elsif ($inheritance eq 'Unknown' || $inheritance eq 'Not applicable' || $inheritance eq 'No data available') {

# do nothing

} else {

$inheritance{$orpha_number} = $inheritance{$orpha_number} . ";" . $inheritance;

}

} else {

$inheritance{$orpha_number} = $inheritance;

}

}

close INHFILE;

# create a hash that has the inheritance groupings

my %inh_classifications = (

'Chromosomal anomaly' => 'Chromosomal anomaly',

'Multigenic/multifactorial;Autosomal dominant;Autosomal recessive' => 'Other multiple forms',

'X-linked dominant;Autosomal dominant;Autosomal recessive' => 'Autosomal & sex-linked',

'Autosomal recessive;Autosomal dominant;Multigenic/multifactorial' => 'Other multiple forms',

'Mitochondrial inheritance;X-linked dominant;Autosomal recessive' => 'Other multiple forms',

'Autosomal dominant;Autosomal recessive;X-linked recessive;Mitochondrial inheritance' => 'Other multiple forms',

'X-linked dominant;Autosomal recessive' => 'Autosomal & sex-linked',

'Autosomal recessive;X-linked recessive;Autosomal dominant' => 'Autosomal & sex-linked',

'X-linked recessive;Autosomal recessive;Autosomal dominant' => 'Autosomal & sex-linked',

'Autosomal dominant;Multigenic/multifactorial;Autosomal recessive' => 'Other multiple forms',

'Autosomal recessive;X-linked recessive;Autosomal dominant;Y-linked' => 'Autosomal & sex-linked',

'X-linked recessive;Autosomal dominant' => 'Autosomal & sex-linked',

'Autosomal dominant;X-linked recessive;Autosomal recessive;Y-linked' => 'Autosomal & sex-linked',

'Mitochondrial inheritance;Autosomal recessive' => 'Other multiple forms',

'Autosomal dominant;X-linked recessive;Autosomal recessive;Mitochondrial inheritance' => 'Other multiple forms',

'X-linked dominant;Autosomal recessive;Autosomal dominant' => 'Autosomal & sex-linked',

'Oligogenic;Autosomal recessive' => 'Other multiple forms',

'Multigenic/multifactorial;Autosomal recessive;Autosomal dominant;X-linked recessive' => 'Other multiple forms',

'Autosomal recessive;X-linked recessive;Autosomal dominant;X-linked dominant' => 'Autosomal & sex-linked',

'X-linked recessive;X-linked dominant' => 'Other multiple forms',

'X-linked recessive;Autosomal dominant;Autosomal recessive' => 'Autosomal & sex-linked',

'X-linked recessive;Autosomal recessive' => 'Autosomal & sex-linked',

'Mitochondrial inheritance;Autosomal dominant;Autosomal recessive' => 'Other multiple forms',

'Autosomal dominant;X-linked recessive;Autosomal recessive' => 'Autosomal & sex-linked',

'Absent' => 'Not applicable',

'Autosomal dominant;Autosomal recessive;X-linked recessive' => 'Autosomal & sex-linked',

'Autosomal recessive;Autosomal dominant' => 'Dominant & recessive, autosomal',

'Autosomal recessive;Autosomal dominant;X-linked recessive' => 'Autosomal & sex-linked',

'Multigenic/multifactorial;Autosomal dominant' => 'Other multiple forms',

'X-linked dominant;Autosomal dominant' => 'Autosomal & sex-linked',

'Multigenic/multifactorial;Autosomal recessive' => 'Other multiple forms',

'X-linked recessive;Autosomal recessive;Autosomal dominant;Mitochondrial inheritance' => 'Other multiple forms',

'Autosomal recessive;X-linked recessive' => 'Autosomal & sex-linked',

'Autosomal dominant;X-linked recessive' => 'Autosomal & sex-linked',

'Autosomal dominant;X-linked dominant' => 'Autosomal & sex-linked',

'Autosomal dominant;Autosomal recessive;X-linked dominant' => 'Autosomal & sex-linked',

'Autosomal recessive;X-linked dominant' => 'Autosomal & sex-linked',

'Autosomal dominant;Autosomal recessive;X-linked recessive;Y-linked' => 'Autosomal & sex-linked',

'Autosomal dominant;Autosomal recessive;X-linked dominant;X-linked recessive' => 'Autosomal & sex-linked',

'Autosomal dominant' => 'Autosomal dominant',

'Autosomal recessive' => 'Autosomal recessive',

'Chromosomal anomaly' => 'Chromosomal anomaly',

'Autosomal dominant;Autosomal recessive' => 'Dominant & recessive, autosomal',

'Mitochondrial inheritance' => 'Mitochondrial',

'Multigenic/multifactorial' => 'Multigenic/multifactorial',

'Unknown' => 'Not applicable',

'Not applicable' => 'Not applicable',

'Not applicable;Unknown' => 'Not applicable',

'No data available' => 'Not applicable',

'Semi-dominant' => 'Other',

'Y-linked' => 'Other',

'Autosomal dominant;Multigenic/multifactorial' => 'Other multiple forms',

'X-linked dominant;X-linked recessive' => 'Other multiple forms',

'Autosomal recessive;Mitochondrial inheritance' => 'Other multiple forms',

'Autosomal dominant;Autosomal recessive;Multigenic/multifactorial' => 'Other multiple forms',

'Autosomal dominant;Autosomal recessive;Mitochondrial inheritance;X-linked recessive' => 'Other multiple forms',

'Autosomal dominant;Mitochondrial inheritance' => 'Other multiple forms',

'Autosomal dominant;Autosomal recessive;Mitochondrial inheritance' => 'Other multiple forms',

'Autosomal dominant;Autosomal recessive;Multigenic/multifactorial;X-linked recessive' => 'Other multiple forms',

'Autosomal recessive;Mitochondrial inheritance;X-linked dominant' => 'Other multiple forms',

'Autosomal recessive;Multigenic/multifactorial' => 'Other multiple forms',

'Autosomal recessive;Oligogenic' => 'Other multiple forms',

'Mitochondrial inheritance;X-linked recessive' => 'Other multiple forms',

'X-linked dominant' => 'X-linked dominant',

'X-linked recessive' => 'X-linked recessive'

);

# create a hash for the group counts

my %group_count;

# create the output files

open (DISOUT, ">disease_output_list.txt");

open (COUNTOUT, ">inheritance_group_counts.txt");

print DISOUT "Orpha_number\tDisorder\tInheritance_patterns\tInheritance_group\n";

# for each of the orpha numbers print out the disease and inheritance patterns

for my $key (keys %disease) {

if (!exists $inheritance{$key}) {

$inheritance{$key} = 'Absent';

}

print DISOUT $key, "\t", $disease{$key}, "\t", $inheritance{$key}, "\t", $inh_classifications{$inheritance{$key}}, "\n";

if (exists $group_count {$inh_classifications{$inheritance{$key}}}) {

$group_count {$inh_classifications{$inheritance{$key}}}++;

} else {

$group_count {$inh_classifications{$inheritance{$key}}} = 1;

}

if (!exists $inh_classifications{$inheritance{$key}}){

print "WARNING ", $inheritance{$key}, " inheritance is not recognised\n";

}

}

for my $group (keys %group_count) {

print COUNTOUT $group, "\t", $group_count{$group}, "\n";

}

close DISOUT;

close COUNTOUT;

# SUBROUTINES

sub dload {

my ($web, $dfile) = @_;

system ("wget ". $web . $dfile);

}

exit;

**######################**

**## Global point prevalence of rare disorders ##**

**######################**

**######################**

**## Exclusion of groups of disorders and disorder sub-types ##**

**######################**

# Go to orphadata.org, epidemiology directory, download file <http://www.orphadata.org/data/xml/en_product9_prv.xml> to desktop and save as xml file.  Open this xml in Excel, and then save as a csv file ‘Rare_diseases_Epidemiology.csv’

######################

## Import data ##

######################

#To import data, follow the instructions below. This will import orphanet rare diseases epidemiological data file assuming that the file is saved as a csv file in your working directory using the name "rd", and that the first row contains variable names.

epi<-read.csv("Rare_diseases_Epidemiology.csv",header=TRUE,sep=";",stringsAsFactors=FALSE,na.strings="")

#Keep relevant variables

rd1<-epi[, c("OrphaNumber","Name","Name6")]

#Rename variable ’Name6’ with 'DisorderType'

rd1$DisorderType<-rd1$Name6

# Exclude groups of disorders and disorders sub-types

disorders0<-rd1[rd1$DisorderType %in% c("Biological anomaly", "Clinical syndrome","Disease","Malformation syndrome","Morphological anomaly","Particular clinical situation in a disease or syndrome"),]

#Remove duplicates

disorders<-disorders0[!duplicated(disorders0$OrphaNumber),]

nrow(disorders)

**######################**

**## Exclusion of rare cancers, infectious diseases and poisonings ##**

**######################**

# First: # Go to orphadata.org, rare disease/classification directory, download file <http://www.orphadata.org/data/xml/en_product3_203.xml> to desktop and save as xml file.  Open this xml in Excel, and then save as a csv file ‘Infectious_RD.csv’

## Import data ##

rid<-

read.csv("Infectious_RD.csv", header=TRUE, sep=";",stringsAsFactors=FALSE,na.strings="")

#Create the list of rare infectious diseases

# Step 1: use of Orphanumbers and Name of diseases

rid1<-rid[,c("OrphaNumber","Name")]

rid2<-rid[,c("OrphaNumber7","Name10")]

rid3<-rid[,c("OrphaNumber14","Name17")]

rid4<-rid[,c("OrphaNumber21","Name24")]

rid5<-rid[,c("OrphaNumber28","Name31")]

rid6<-rid[,c("OrphaNumber35","Name38")]

#remove duplicate entities

rid1s<- rid1 [!duplicated(rid1$OrphaNumber),]

rid2s<- rid2 [!duplicated(rid2$OrphaNumber7),]

rid3s<- rid3 [!duplicated(rid3$OrphaNumber14),]

rid4s<- rid4 [!duplicated(rid4$OrphaNumber21),]

rid5s<- rid5 [!duplicated(rid5$OrphaNumber28),]

rid6s<- rid6 [!duplicated(rid6$OrphaNumber35),]

# rename variables

rid2s $ OrphaNumber <- rid2s $ OrphaNumber7

rid3s $ OrphaNumber <- rid3s $ OrphaNumber14

rid4s $ OrphaNumber <- rid4s $ OrphaNumber21

rid5s $ OrphaNumber <- rid5s $ OrphaNumber28

rid6s $ OrphaNumber <- rid6s $ OrphaNumber35

rid2s $ Name <- rid2s $ Name10

rid3s $ Name <- rid3s $ Name17

rid4s $ Name <- rid4s $ Name24

rid5s $ Name <- rid5s $ Name31

rid6s $ Name <- rid6s $ Name38

#Keep relevant variables

rid2ss<-rid2s[,c("OrphaNumber","Name")]

rid3ss<-rid3s[,c("OrphaNumber","Name")]

rid4ss<-rid4s[,c("OrphaNumber","Name")]

rid5ss<-rid5s[,c("OrphaNumber","Name")]

rid6ss<-rid6s[,c("OrphaNumber","Name")]

# list of rare infectious diseases

infectious<-rbind(rid1s,rid2ss, rid3ss, rid4ss, rid5ss, rid6ss)

infectious<-infectious[!duplicated(infectious$OrphaNumber),]

#Merge the table of rare diseases with the table of rare disorders

rd_inf<-merge(disorders,infectious,by="OrphaNumber",all.x=T)

# Keep rare infectious disorders

INFECTIOUS <-rd_inf[which(!is.na(rd_inf$Name.y)),]

#calculate the proportion of rare infectious disorders

nrow(INFECTIOUS)/nrow(disorders)*100

# Create a new variable “Classification”

INFECTIOUS$Classification<-"Infect"

##################################

# Second: Go to orphadata.org, rare disease/classification directory, download file <http://www.orphadata.org/data/xml/en_product3_202.xml> to desktop and save as xml file.  Open this xml in Excel, and then save as a csv file ‘Neoplastic_RD.csv’

## Import data ##

rcd<-

read.csv("Neoplastic_RD.csv", header=TRUE,sep=";",stringsAsFactors=FALSE,na.strings="")

#Create the list of rare neoplastic diseases

# Step 1: use of Orphanumbers and Name of diseases

rcd1<-rcd[,c("OrphaNumber","Name")]

rcd2<-rcd[,c("OrphaNumber7","Name10")]

rcd3<-rcd[,c("OrphaNumber14","Name17")]

rcd4<-rcd[,c("OrphaNumber21","Name24")]

rcd5<-rcd[,c("OrphaNumber28","Name31")]

rcd6<-rcd[,c("OrphaNumber35","Name38")]

rcd7<-rcd[,c("OrphaNumber42","Name45")]

rcd8<-rcd[,c("OrphaNumber49","Name52")]

rcd9<-rcd[,c("OrphaNumber56","Name59")]

rcd10<-rcd[,c("OrphaNumber63","Name66")]

rcd11<-rcd[,c("OrphaNumber70","Name73")]

#remove duplicate entities

rcd1s<- rcd1 [!duplicated(rcd1$OrphaNumber),]

rcd2s<- rcd2 [!duplicated(rcd2$OrphaNumber7),]

rcd3s<- rcd3 [!duplicated(rcd3$OrphaNumber14),]

rcd4s<- rcd4 [!duplicated(rcd4$OrphaNumber21),]

rcd5s<- rcd5 [!duplicated(rcd5$OrphaNumber28),]

rcd6s<- rcd6 [!duplicated(rcd6$OrphaNumber35),]

rcd7s<- rcd7 [!duplicated(rcd7$OrphaNumber42),]

rcd8s<- rcd8 [!duplicated(rcd8$OrphaNumber49),]

rcd9s<- rcd9 [!duplicated(rcd9$OrphaNumber56),]

rcd10s<- rcd10 [!duplicated(rcd10$OrphaNumber63),]

rcd11s<- rcd11 [!duplicated(rcd11$OrphaNumber70),]

# rename variables

rcd2s $ OrphaNumber <- rcd2s $ OrphaNumber7

rcd3s $ OrphaNumber <- rcd3s $ OrphaNumber14

rcd4s $ OrphaNumber <- rcd4s $ OrphaNumber21

rcd5s $ OrphaNumber <- rcd5s $ OrphaNumber28

rcd6s $ OrphaNumber <- rcd6s $ OrphaNumber35

rcd7s $ OrphaNumber <- rcd7s $ OrphaNumber42

rcd8s $ OrphaNumber <- rcd8s $ OrphaNumber49

rcd9s $ OrphaNumber <- rcd9s $ OrphaNumber56

rcd10s $ OrphaNumber <- rcd10s $ OrphaNumber63

rcd11s $ OrphaNumber <- rcd11s $ OrphaNumber70

rcd2s $ Name <- rcd2s $ Name10

rcd3s $ Name <- rcd3s $ Name17

rcd4s $ Name <- rcd4s $ Name24

rcd5s $ Name <- rcd5s $ Name31

rcd6s $ Name <- rcd6s $ Name38

rcd7s $ Name <- rcd7s $ Name45

rcd8s $ Name <- rcd8s $ Name52

rcd9s $ Name <- rcd9s $ Name59

rcd10s $ Name <- rcd10s $ Name66

rcd11s $ Name <- rcd11s $ Name73

#Keep relevant variables

rcd2ss<-rcd2s[,c("OrphaNumber","Name")]

rcd3ss<-rcd3s[,c("OrphaNumber","Name")]

rcd4ss<-rcd4s[,c("OrphaNumber","Name")]

rcd5ss<-rcd5s[,c("OrphaNumber","Name")]

rcd6ss<-rcd6s[,c("OrphaNumber","Name")]

rcd7ss<-rcd7s[,c("OrphaNumber","Name")]

rcd8ss<-rcd8s[,c("OrphaNumber","Name")]

rcd9ss<-rcd9s[,c("OrphaNumber","Name")]

rcd10ss<-rcd10s[,c("OrphaNumber","Name")]

rcd11ss<-rcd11s[,c("OrphaNumber","Name")]

# list of rare neoplastic diseases

cancer<-rbind(rcd1s,rcd2ss, rcd3ss, rcd4ss, rcd5ss, rcd6ss, rcd7ss, rcd8ss, rcd9ss, rcd10ss, rcd11ss)

cancer<-cancer [!duplicated(cancer$OrphaNumber),]

# Merge the table of rare neoplastic diseases with the table of rare disorders

rd_can<-merge(disorders,cancer,by="OrphaNumber",all.x=T)

# Keep rare neoplastic disorders

CANCERS<-rd_can[which(!is.na(rd_can$Name.y)),]

#Total number of rare neoplastic disorders

nrow(CANCERS)

#calculate the proportion of rare neoplastic disorders

nrow(CANCERS)/nrow(disorders)*100

# Create a new variable “Classification”

CANCERS$Classification <-"Cancer"

##################################

# Third: Go to orphadata.org, rare disease/classification directory, download file <http://www.orphadata.org/data/xml/en_product3_204.xml> to desktop and save as xml file.  Open this xml in Excel, and then save as a csv file ‘Intoxication_RD.csv’

## Import data ##

int<-read.csv("Intoxication_RD.csv", header=TRUE,sep=";",stringsAsFactors=FALSE,na.strings="")

# Step 1: use of Orphanumbers and Name of diseases

int1<-int[,c("OrphaNumber","Name")]

int2<-int[,c("OrphaNumber7","Name10")]

int3<-int[,c("OrphaNumber14","Name17")]

int4<-int[,c("OrphaNumber21","Name24")]

#remove duplicate entities

int1s<- int1 [!duplicated(int1$OrphaNumber),]

int2s<- int2 [!duplicated(int2$OrphaNumber7),]

int3s<- int3 [!duplicated(int3$OrphaNumber14),]

int4s<- int4 [!duplicated(int4$OrphaNumber21),]

# rename variables

int2s $ OrphaNumber <- int2s $ OrphaNumber7

int3s $ OrphaNumber <- int3s $ OrphaNumber14

int4s $ OrphaNumber <- int4s $ OrphaNumber21

int2s $ Name <- int2s $ Name10

int3s $ Name <- int3s $ Name17

int4s $ Name <- int4s $ Name24

#Keep relevant variables

int2ss<-int2s[,c("OrphaNumber","Name")]

int3ss<-int3s[,c("OrphaNumber","Name")]

int4ss<-int4s[,c("OrphaNumber","Name")]

# list of poisonings

intox<-rbind(int1s,int2ss, int3ss, int4ss)

intox<-intox [!duplicated(intox$OrphaNumber),]

# Merge table of poisonings with the table of rare disorders

rd_intox<-merge(disorders,intox,by="OrphaNumber",all.x=T)

# Keep poisonings

INTOXICATIONS<-rd_intox[which(!is.na(rd_intox$Name.y)),]

#calculate the proportion of poisonings

nrow(INTOXICATIONS)/nrow(disorders)*100

#Total number of poisonings

nrow(INTOXICATIONS)

# Create a new variable “Classification”

INTOXICATIONS$Classification <-"Intox"

##################################

#Combine tables of rare cancers, infectious diseases and poisonings

cancer_infect<-rbind(CANCERS,INFECTIOUS)

cancer_infect_intox<-rbind(cancer_infect,INTOXICATIONS)

# remove duplicates

exclus0<- cancer_infect_intox [!duplicated(cancer_infect_intox $OrphaNumber),]

# Take look at the number of rare disorder excluded from each classification

table(exclus0$Classification)

##################################

# Merge the new table with the table of rare disorders

rd_all<-merge(disorders,exclus0,by="OrphaNumber",all.x=T)

# Exclude rare cancers, infectious diseases and poisonings

rd_inclus<-rd_all[which(is.na(rd_all$Classification)),]

#Keep the relevant variables

rd_in<-rd_inclus[,c("OrphaNumber","Name")]

#Number of prevalent rare disorders

nrow(rd_in)

##################################

**######################**

**## Use of Orphanet epidemiological data file ##**

**######################**

# Go to orphadata.org, epidemiology directory, download file <http://www.orphadata.org/data/xml/en_product9_prv.xml> to desktop and save as xml file.  Open this xml in Excel, and then save as a csv file ‘Rare_diseases_Epidemiology.csv’

######################

## Import data ##

######################

epi<-read.csv("Rare_diseases_Epidemiology.csv",header=TRUE,sep=";",stringsAsFactors=FALSE,na.strings="", ,dec=",")

#Keep the relevant variables

epi1<-epi[, c("OrphaNumber","Name","Name12","Name16","Name20","ValMoy","Name24")]

#Rename variables

#TypTag for the type of epidemiological indicator

#SubTag for the subtype of epidemiological indicator

#SubTag for the prevalence class

#GeoTag for the geographical area

epi1$TypTag <- epi1$Name12

epi1$SubTag <- epi1$Name16

epi1$ClsTag <- epi1$Name20

epi1$GeoTag <- epi1$Name24

epi2<- epi1[, c("OrphaNumber","Name","TypTag","SubTag","ClsTag","ValMoy","GeoTag")]

# Merge the table of epidemiological data with the table of prevalent rare disorders

rd_in_epi<-merge(rd_in,epi2,by="OrphaNumber",all.x=T)

# Remove duplicates

rdsd<-rd_in_epi[!duplicated(rd_in_epi$OrphaNumber),]

#Number of rare disorders described with at list one epidemiological indicator

rdsd0<-rdsd[!duplicated(rdsd$OrphaNumber),]

## Number of rare disorders that are not yet annotated

nrow(disorders)-nrow(rdsd0)

#Keep rare disorders described by "point prevalence" and "cases/families"

rd_prv<-rd_in_epi[rd_in_epi$TypTag %in% c("Point prevalence", "Cases/families"),]

rd_prv0<- rd_prv[!duplicated(rd_prv$OrphaNumber),]

#Remove rare disorders with unknown prevalence

rd_prvC<- rd_prv[rd_prv $ClsTag %in% c("<1 / 1 000 000","1-9 / 1 000 000","1-9 / 100 000","1-5 / 10 000","6-9 / 10 000",">1 / 1000" ),]

##Remove duplicate entities

rd_prvCsd<- rd_prvC[!duplicated(rd_prvC$ OrphaNumber),]

# Number of rare disorders described by the relevant epidemiological indicator

nrow(rd_prvCsd)

**# List of rare disorders described by a number of cases or families**

rd_CF<- rd_prv [rd_prv$SubTag %in% c("Case","Family"),]

#Number of rare disorders described by a number of cases or families

nrow(rd_CF)

#Take look at the table

table(rd_CF$SubTag)

#Create a new variable ‘CasFam’

rd_CF$CasFam<-"CF"

#Keep relevant variables

rd_CF0<-rd_CF[,c("OrphaNumber","CasFam")]

**######################**

**##Calculation of Indirect point prevalence estimates ##**

**######################**

#Create two tables: the first with the number of "cases" and the second with the number of "families"

RDs_cases<- rd_prv [rd_prv$SubTag== "Case",]

RDs_families<- rd_prv [rd_prv$SubTag== "Family",]

# When considering the global population size at the year 2017 ~ 7.550.000.000)

**# Indirect point prevalence estimates**

# This assumes that one family report has equal weighting to one case report

(sum(RDs_cases$ValMoy)+(sum(RDs_families$ValMoy)*1))/7550000000

#Results: 1.180106e-05

# Use 10 cases per family instead of one case per family.

(sum(RDs_cases$ValMoy)+(sum(RDs_families$ValMoy)*10))/7550000000

#Results: 1.58755e-05

**######################**

**## Calculation of overall point prevalence estimates##**

**######################**

**# STEP1: Use the list of rare disorders described by numerical point prevalence value**

RDs_preval<-rd_prv[rd_prv$TypTag== "Point prevalence",]

#Remove rare disorders with unknown prevalence

RDs_prvevalC0<- RDs_preval [RDs_preval $ClsTag %in% c("<1 / 1 000 000","1-9 / 1 000 000","1-9 / 100 000","1-5 / 10 000","6-9 / 10 000",">1 / 1000" ),]

# Remove rare disorders described by prevalence in particular population (Not defined geographical area)

RDs_prvevalC1<- RDs_prvevalC0 [RDs_prvevalC0$GeoTag!="Not defined",]

# Exclude rare disorders already described by cases or families

CF_preval<-merge(rd_CF0, RDs_prvevalC1,by="OrphaNumber",all.y=T)

preval<-CF_preval [which(is.na(CF_preval$CasFam)),]

table(preval$ClsTag)

prevalsd<-preval[!duplicated(preval$OrphaNumber),]

nrow(prevalsd)

# Selection on geographical area

#Select Worldwide prevalence (preval0_W)

preval0_W<-preval[preval$GeoTag=="Worldwide",]

preval0_W0<-preval0_W[preval0_Eu$ClsTag %in% c("<1 / 1 000 000","1-9 / 1 000 000","1-9 / 100 000","1-5 / 10 000"),]

# Select Europe prevalence (preval0_E)

preval0_Eu<-preval[preval$GeoTag=="Europe",]

preval0_E<-preval0_Eu[preval0_Eu$ClsTag %in% c("<1 / 1 000 000","1-9 / 1 000 000","1-9 / 100 000","1-5 / 10 000"),]

# Select USA prevalence (preval0_U)

preval0_Us<-preval[preval$GeoTag=="United States",]

preval0_U<-preval0_Us[preval0_Us$ClsTag %in% c("<1 / 1 000 000","1-9 / 1 000 000","1-9 / 100 000","1-5 / 10 000"),]

#Merge tables of Worlwide and Europe then select those which are only available in Europe

prevalWE<-merge(preval0_W,preval0_E,by="OrphaNumber",all=F)

prevalWE$WE<-"A"

prevalWE0<-prevalWE[,c("OrphaNumber","WE")]

preval0_E0<-merge(preval0_E,prevalWE0,by="OrphaNumber",all=T)

preval_UEs<-preval0_E0[which(is.na(preval0_E0$WE)),]

# Merge tables of Worlwide and USA then select those which are only available in the USA

prevalWU<-merge(preval0_W,preval0_U,by="OrphaNumber",all=F)

prevalWU$WU<-"B"

prevalWU0<-prevalWU[,c("OrphaNumber","WU")]

preval0_U0<-merge(preval0_U,prevalWU0,by="OrphaNumber",all=T)

preval_Us<-preval0_U0[which(is.na(preval0_U0$WU)),]

# Merge tables of Europe and USA then select those which are only available in the USA

prevalEU<-merge(preval_UEs,preval_Us,by="OrphaNumber",all=F)

prevalEU$EU<-"C"

#Keep relevant variables

prevalEU0<-prevalEU[,c("OrphaNumber","EU")]

preval_U1<-merge(preval_Us,prevalEU0,by="OrphaNumber",all=T)

preval_U1s<-preval_U1[which(is.na(preval_U1$EU)),]

#### Point prevalence table (worldwide/Europe/USA)

### Keep relevant variables

prv_W<-preval0_W[,c("OrphaNumber","Name.x","TypTag","SubTag","ClsTag","ValMoy","GeoTag")]

prv_E<-preval_UEs[,c("OrphaNumber","Name.x","TypTag","SubTag","ClsTag","ValMoy","GeoTag")]

prv_U<-preval_U1s[,c("OrphaNumber","Name.x","TypTag","SubTag","ClsTag","ValMoy","GeoTag")]

#Merge the previous tables

prv_WE<-rbind(prv_W,prv_E)

prv_WEU<-rbind(prv_WE,prv_U)

table (prv_WEU$GeoTag)

prv_WEUF<-prv_WEU[prv_WEU$ClsTag %in% c("<1 / 1 000 000","1-9 / 1 000 000","1-9 / 100 000","1-5 / 10 000"),]

#Number of rare disorders that are included

nrow(prv_WEUF)

#Cumulative prevalence using only point prevalence numerical values

sum(prv_WEUF$ValMoy)

##Results: cumulative point prevalence of 2776.994 per 100,000 using point prevalence value only of 724 RDs.

**#STEP2: Use the list of rare disorders described by point prevalence range only**

## Use of minimum boundaries

table(prv_WEUF$ClsTag)

## select rare disorders with point prevalence "class only"

prv_WEU_cl<-prv_WEUF[prv_WEU$SubTag=="Class only" ,]

prv_WEU_valcl<-prv_WEUF[prv_WEU$SubTag=="Value and class" ,]

table (prv_WEU_valcl$ClsTag)

nrow(prv_WEU_valcl)

# remove the variable "ValMoy"

prv_WEU_cl<-prv_WEU_cl[,c("OrphaNumber","Name.x","TypTag","SubTag","ClsTag","GeoTag")]

#Take look to the table

table(prv_WEU_cl$ClsTag)

## Create a new variable with the minimun boundaries

#prv_WEU_cl0<-prv_WEU[prv_WEU$SubTag=="Class only" & prv_WEU$ClsTag=="<1 / 1 000 000",]

#Change "<1 / 1 000 000" class to 0.1 per 100 000

prv_WEU_cl0<-prv_WEU[prv_WEU$SubTag=="Class only" & prv_WEU$ClsTag=="<1 / 1 000 000",]

prv_WEU_cl0$ValMoy<-ifelse(prv_WEU_cl0$ClsTag=="<1 / 1 000 000","0.10","0.0")

prv_WEU_cl0$ValMoy<-as.numeric(prv_WEU_cl0$ValMoy)

sum(prv_WEU_cl0$ValMoy)

## Change "1-5 / 10 000" class to 10 per 100 000

prv_WEU_cl1<-prv_WEU_cl[prv_WEU_cl$ClsTag=="1-5 / 10 000",]

prv_WEU_cl1$ValMoy<-ifelse(prv_WEU_cl1$ClsTag=="1-5 / 10 000","10.00","0")

prv_WEU_cl1$ValMoy<-as.numeric(prv_WEU_cl1$ValMoy)

sum(prv_WEU_cl1$ValMoy)

##Change "1-9 / 100 000" class to 1 per 100 000

prv_WEU_cl2<-prv_WEU_cl[prv_WEU_cl$SubTag=="Class only" & prv_WEU_cl$ClsTag== "1-9 / 100 000", ]

prv_WEU_cl2$ValMoy<-ifelse(prv_WEU_cl2$ClsTag== "1-9 / 100 000","1.00","0")

prv_WEU_cl2$ValMoy<-as.numeric(prv_WEU_cl2$ValMoy)

sum(prv_WEU_cl2$ValMoy)

##Change "1-9 / 1 000 000" class to 0.1 per 100 000

prv_WEU_cl3<-prv_WEU_cl[prv_WEU_cl$SubTag=="Class only" & prv_WEU_cl$ClsTag== "1-9 / 1 000 000",]

prv_WEU_cl3$ValMoy<-ifelse(prv_WEU_cl3$ClsTag== "1-9 / 1 000 000","0.10","0")

prv_WEU_cl3$ValMoy<-as.numeric(prv_WEU_cl3$ValMoy)

sum(prv_WEU_cl3$ValMoy)

# Combine all the tables of values

prvc1<-rbind(prv_WEU_cl1,prv_WEU_cl2)

prvc2<-rbind(prvc1,prv_WEU_cl0)

prvcl<-rbind(prvc2,prv_WEU_cl3)

sum(prvcl$ValMoy)

nrow(prvcl)

##Results: Cumulative point prevalence of 540.9 per 100,000 using minimum boundaries of 360 RDs.

# Use of maximum boundaries

PRV_WEU_cl<-prv_WEU_cl[,c("OrphaNumber","Name.x","TypTag","SubTag","ClsTag","GeoTag")]

# Modification of mean values according to prevalence class

# remove the variable "ValMoy"

PRV_WEU_cl<-PRV_WEU_cl[,c("OrphaNumber","Name.x","TypTag","SubTag","ClsTag","GeoTag")]

table(PRV_WEU_cl$ClsTag)

## Create a new variable with the maximum value

##Change "<1 / 1 000 000" class to 0.1 per 100 000

PRV_WEU_cl0<-prv_WEU[prv_WEU$SubTag=="Class only" & prv_WEU$ClsTag=="<1 / 1 000 000",]

PRV_WEU_cl0$ValMoy<-ifelse(PRV_WEU_cl0$ClsTag=="<1 / 1 000 000","0.1","0")

PRV_WEU_cl0$ValMoy<-as.numeric(PRV_WEU_cl0$ValMoy)

sum(PRV_WEU_cl0$ValMoy)

#Change "1-5 / 10 000"class to 50 per 100 000

PRV_WEU_cl1<-prv_WEU_cl[prv_WEU_cl$ClsTag=="1-5 / 10 000",]

PRV_WEU_cl1$ValMoy<-ifelse(PRV_WEU_cl1$ClsTag=="1-5 / 10 000","50.00","0")

PRV_WEU_cl1$ValMoy<-as.numeric(PRV_WEU_cl1$ValMoy)

sum(PRV_WEU_cl1$ValMoy)

#Change"1-9 / 100 000" class to 9 per 100 000

PRV_WEU_cl2<-prv_WEU_cl[prv_WEU_cl$SubTag=="Class only" & prv_WEU_cl$ClsTag== "1-9 / 100 000", ]

PRV_WEU_cl2$ValMoy<-ifelse(PRV_WEU_cl2$ClsTag== "1-9 / 100 000","9.00","0")

PRV_WEU_cl2$ValMoy<-as.numeric(PRV_WEU_cl2$ValMoy)

sum(PRV_WEU_cl2$ValMoy)

#Change"1-9 / 1 000 000" class to 0.9 per 100 000

PRV_WEU_cl3<-prv_WEU_cl[prv_WEU_cl$SubTag=="Class only" & prv_WEU_cl$ClsTag== "1-9 / 1 000 000",]

PRV_WEU_cl3$ValMoy<-ifelse(PRV_WEU_cl3$ClsTag== "1-9 / 1 000 000","0.90","0")

PRV_WEU_cl3$ValMoy<-as.numeric(PRV_WEU_cl3$ValMoy)

sum(PRV_WEU_cl3$ValMoy)

# Combine all the tables of values

PRVc1<-rbind(PRV_WEU_cl1,PRV_WEU_cl2)

PRVc2<-rbind(PRVc1,PRV_WEU_cl0)

PRVcl<-rbind(PRVc2,PRV_WEU_cl3)

sum(PRVcl$ValMoy)

nrow(PRVcl)

#Results: Cumulative point prevalence of 2968.9 per 100,000 using maximum boundary of 360 RDs.

**#STEP3: Use of point prevalence in European countries**

# Create the list of rare disorders for which data were used

# Table of rare disorders described by a number of cases or families

rd_CF0$WEU<-"WEU"

RDCF<-rd_CF0[,c(1,3)]

# Table of rare disorders described by prevalence range only

PRVcl$WEU<-"WEU"

PRVcol<-PRVcl[,c(1,8)]

# Table of rare disorders described by prevalence numerical values

prv_WEUF$WEU<-"WEU"

PRVal<-prv_WEUF[,c(1,8)]

# combine tables

RD_data<-rbind(RDCF,PRVcol,PRVal)

nrow(RD_data)

## Disorders with data on geographical area which is not "Worldwide","Europe"and "United States"

# Exclude the previous disorders

preval_con<-merge(RD_data,rd_prv,by="OrphaNumber",all.y=T)

preval_con_only<-preval_con[which(is.na(preval_con$WEU)),]

table(preval_con_only$SubTag)

nrow(preval_con_only)

# Keep disorders described by a prevalence numerical value

preval_con_only0<-preval_con_only[preval_con_only$SubTag=="Value and class",]

nrow(preval_con_only0)

## Remove data on particular population

preval_con_only1<-preval_con_only0[preval_con_only0$GeoTag!="Not defined",]

nrow(preval_con_only1)

table(preval_con_only1$GeoTag)

## Number of disorders with data on geographical area different to "worldwide","europe"and "united States"

preval_con_only2<-preval_con_only1[!duplicated(preval_con_only0$OrphaNumber),]

nrow(preval_con_only2)

# Keep european countries for which prevalence value are available

preval_con_only10<-preval_con_only1[preval_con_only1$ValMoy!=0,]

preval_con_only11<-preval_con_only10[preval_con_only10$GeoTag %in% c("Finland","United Kingdom","Italy","Norway","New Zealand","Portugal","Ireland",'Greece',"Spain","Sweden","France","Iceland","Denmark","Cyprus","Czech Republic","Netherlands"),]

preval_con_only12<-preval_con_only11[!duplicated(preval_con_only11$OrphaNumber),]

# Calculate the mean prevalence estimates

#Preval_con_agg_mean<-aggregate(preval_con_only11$ValMoy,by=list(preval_con_only11$OrphaNumber),FUN=mean,na.rm=T)

Preval_con_agg_mean<-aggregate(ValMoy~OrphaNumber+Name.x,preval_con_only11,FUN=mean,na.rm=T)

# Exclude mean prevalence >50/100,000

Preval_con_agg_mean1<-Preval_con_agg_mean[Preval_con_agg_mean$ValMoy<50,]

nrow(Preval_con_agg_mean1)

#Calculate the cumulative prevalence using mean prevalence value of european countries only

prvcon<-sum(Preval_con_agg_mean1$ValMoy)

prvcon

**#############################**

**# STEP 4: Calculation of globale cumulative point prevalence estimates**

**################################**

**## Minimum boundary**

Preval_global<-sum(prvcon,sum(prv_WEU$ValMoy),sum(prvcl$ValMoy),((sum(RDs_cases$ValMoy)+(sum(RDs_families$ValMoy)*1))/7550000000))

Preval_global

# results:3482.284

**### Maximum boundary**

PREVAL_global<-sum(prvcon,sum(prv_WEU$ValMoy),sum(PRVcl$ValMoy),((sum(RDs_cases$ValMoy)+(sum(RDs_families$ValMoy)*1))/7550000000))

PREVAL_global # results:5910.284

**######################**

**## Values used for tables and figures**

**######################**

**## Distribution of rare disorders and rare disorders patients number##**

**######################**

##Number of rare disorders described by cases and families reports (point prevalence class of "<1/1 000 000")

nrow(rd_CF)

#2840

# Number of rare disorders described by ranges

nrow(prvcl)

#724

table(prvcl$ClsTag)

#<1 / 1 000 000 1-5 / 10 000 1-9 / 1 000 000 1-9 / 100 000

#190 145 154 234

# Number of rare disorders described by the prevalence class of "<1/1 000 000"

nrow(prv_WEU_cl0)

# 174

# Number of rare disorders described by a mean prevalence value

nrow(preval_con_only12)

#<1 / 1 000 000 1-5 / 10 000 1-9 / 1 000 000 1-9 / 100 000

#1 4 9 7

# Total number of RDs by prevalence class

#class "<1/1 000 000" (3031 Rds)

2840+190+1

#class "1-9 / 1 000 000"(164 Rds)

155+9

#class "1-9 / 100 000" (241 Rds)

234+7

#class "1-5 / 10 000" (149 Rds)

145+4

**#Total number of rare disorders which are included (RDs=3585)**

Class <- c("<1/1 000 000","1-9/1 000 000","1-9/100 000","1-5/10 000")

total <- c(3031,164,241,149)

somme <- sum(total[1:4]);somme

total_s <-c(3031,164,241,149)/somme*100

total_s

# Distribution by ranges (results)

#"<1/1 000 000","1-9/1 000 000","1-9/100 000","1-5/10 000"

# 84.546722 4.574616 6.722455 4.156206

**# Distribution of RD patients by prevalence class**

**# Use the minimum boundary (per 100,000)**

Class1 <- c("<1/1 000 000","1-9/1 000 000","1-9/100 000","1-5/10 000")

total1 <- c((3031*0.1),(164*0.1),(241*1),(149*10))

somme_min <- sum(total1[1:4]);somme_min

total_smin <-c((3031*0.1),(164*0.1),(241*1),(149*10))/somme_min*100;total_smin

# Distribution of RD patients by prevalence class using the minimum boundary

#"<1/1 000 000","1-9/1 000 000","1-9/100 000","1-5/10 000"

# 14.7817605 0.7998049 11.7532309 72.6652036

**# Use the maximum boundary (per 100,000)**

Class2 <- c("<1/1 000 000","1-9/1 000 000","1-9/100 000","1-5/10 000")

total2 <- c((3031*0.1),(164*0.9),(241*9),(149*50))

somme_max <- sum(total2[1:4]);somme_max

total_smax <-c((3031*0.1),(164*0.9),(241*9),(149*50))/somme_max*100;total_smax

# Distribution of RD patients by prevalence class using the maximum boundary

#"<1/1 000 000","1-9/1 000 000","1-9/100 000","1-5/10 000"

# 3.010020 1.465783 21.539867 73.984329
